# Tumor stroma Siglec15 expression is a poor prognosis predictor in colon adenocarcinoma

**DOI:** 10.7150/jca.87618

**Published:** 2023-09-18

**Authors:** Weixiang Zhan, Fan Bai, Yue Cai, Jianwei Zhang, Ge Qin, Yuqian Xie, Yanhong Deng

**Affiliations:** 1 Department of Oncology, The Sixth Affiliated Hospital, Sun Yat-sen University, Guangzhou, China.; 2 Guangdong Provincial Key Laboratory of Colorectal and Pelvic Floor Disease, Guangdong Research Institute of Gastroenterology, The Sixth Affiliated Hospital, Sun Yat-sen University, Guangzhou, China.; 3 Biomedical Innovation Center, The Sixth Affiliated Hospital, Sun Yat-sen University, Guangzhou, China.

**Keywords:** Siglec15, PD-L1, immune checkpoint, immunotherapy, colon adenocarcinoma

## Abstract

Sialic acid binding Ig-like lectin 15 (Siglec15) is considered a novel immune checkpoint and an emerging target for next-generation cancer immunotherapy. However, the significance of Siglec15 and its relationship with programmed death-ligand 1 (PD-L1) in colon adenocarcinoma (COAD) remain unknown. In this study, we analyzed Siglec15 expression within stromal area (SA) and tumor area (TA), and its relationship with tumor-infiltrating lymphocytes (TILs) in COAD and mismatch repair-proficient (MMR-p) COAD. Siglec15 expression was significantly higher in COAD tissues than in normal tissues, and elevated Siglec15(SA) expression, rather than Siglec15(TA) and Siglec15 (whole) expression, was correlated with poor prognosis and inversely correlated with the density of CD8^+^ T cell, both in COAD and MMR-p COAD. Moreover, there were no correlations between Siglec15(SA) and PD-L1(SA), and between Siglec15(TA) and PD-L1(TA), whereas there was positive correlation between Siglec15(whole) and PD-L1(whole). A new immune classification based on the Siglec15(SA)/PD-L1(SA) expression, indicated that patients with Siglec15(SA)^Low^/PD-L1(SA)^+^ status had the longest survival times in COAD. Our study highlights that Siglec15(SA) is an independent predictor of poor prognosis and has an immunosuppressive role in COAD and MMR-p COAD tissues. These findings may provide insights into improving responses to immunotherapy-included comprehensive treatments for COAD in the future.

## Introduction

Colorectal cancer (CRC) is the third most common cancer and second most frequent cause of cancer-related deaths worldwide^1^. CRC is classified into two major groups: mismatch repair-deficient (MMR-d) and mismatch repair-proficient (MMR-p). Treatments of CRC include surgery, chemotherapy, radiotherapy and emerging immunotherapy^2^. Immune checkpoint inhibitors (ICIs) targeting programmed death-1 (PD-1) and anti-programmed cell death ligand 1 (PD-L1) have exhibited a durable response and currently dominate the method of treatment for various tumor types^3^-^5^. However, anti-PD-1/PD-L1 therapy is considered ineffective in most patients with CRC, and only those with MMR-d tumors and a high tumor mutational burden (TMB) have been found to be responsive to anti-PD-1/PD-L1 therapy^6^-^9^. Most CRC patients with MMR-p, which comprise approximately 95% of metastatic CRC cases, often do not benefit from current immunotherapy approaches. Thus, a predictive immune biomarker is urgently needed to be identified, especially in MMR-p CRC.

Sialic acid-binding immunoglobulin-like lectin 15 (Siglec15), a member of the sialic acid-binding immunoglobulin-like lectin family, is one of the most evolutionarily conserved Siglecs in vertebrates and is phylogenetically distant from other family members^10^. Siglec15 is originally found to be overexpressed in giant cell tumors of the bone and was discovered to regulate osteoclast differentiation and bone remodeling through interaction with the signaling adaptor DAP12^11^-^14^. Recent research has found that Siglec15 is also broadly upregulated in human tumor area and tumor infiltrating myeloid cells, and its expression is mutually exclusive to PD-L1. Siglec15 is considered a novel anti-tumor target comparable to PD-L1, and has the ability to sustainably suppress T-cell responses and elicit immune evasion in the tumor microenvironment^15^. Therefore, Siglec15 is considered a promising new target for normalization cancer immunotherapy independent of the PD-1/PD-L1 pathway, and targeting Siglec15 may be an effective alternative therapy for patients who do not respond to PD-1/PD-L1 antibodies 16-18.

However, the significance of Siglec15 in cancer is uncertain. High Siglec15 levels are associated with poor prognosis in lung adenocarcinoma (LUAD) and kidney cancer, but are associated with better prognoses in bladder urothelial carcinoma (BLCA), breast invasive carcinoma (BRCA), head and neck squamous cell carcinoma (HNSC), thyroid cancer (THCA) and uterine corpus endometrial carcinoma (UCEC) 19-23. The relationship between Siglec15 and the immune microenvironment in colon adenocarcinoma (COAD), especially MMR-p COAD, has not been elucidated.

Moreover, PD-L1 expression is related to T-cell subpopulations in CRC immune microenvironment, and the PD-1/PD-L1 axis is considered a clinically relevant mediator of tumor immune escape^24^-^26^. Meanwhile, PD-L1 can enable tumor cells to evade immune elimination by negatively regulating T-cell immune responses^27^. The relationship between Siglec15 and PD-L1 in COAD remains unclear.

Here, we evaluated the relationship between Siglec15 expression (in the stromal area (SA), tumor area (TA) or whole cells), clinicopathological characteristics, and the immune microenvironment in patients with COAD. We found that Siglec15 was significantly higher expression in COAD than normal intestinal tissue, and Siglec15(SA)^High^, rather than Siglec15(TA)^High^ and Siglec15(whole)^High^, correlated with poor prognosis. Notably, Siglec15(SA) served as an immune suppressor by suppressing CD8^+^ T cell, but not CD4^+^ T cell in COAD and MMR-p COAD. Besides, there were no correlations between Siglec15(SA) and PD-L1(SA), and between Siglec15(TA) and PD-L1(TA), whereas there was positive correlation between Siglec15(whole) and PD-L1(whole). A new immune classification, based on the expressions of Siglec15(SA)/PD-L1(SA), indicating that patients with Siglec15(SA)^Low^/PD-L1(SA)^+^ status had the longest survival. Our study may provide new insights into patients with COAD or MMR-p COAD to improve the ability to select the appropriate immunotherapy.

## Material and methods

### Patients and samples

Our clinical cohort was based on a tissue microarray (TMA) purchased from Shanghai Outdo Biotech. Patients were eligible for inclusion if they had histologically confirmed colon adenocarcinoma, and without any previous systemic anticancer therapy for colon cancer disease, and underwent initial resections. Paraffin-embedded pathological specimens were obtained from 102 patients with COAD, including 76 matched adjacent normal tissues. Patients were collected from July 2006 to May 2007, with 10-year follow-ups. Clinicopathological variables included general information, tumor location, tumor growth pattern, degree of differentiation, depth of tumor invasion, nodal status, metastatic status, mismatch repair (MMR) status, and the outcome of follow-up data. Tumor pT, pN, and pM statuses were assessed according to the criteria of the Seventh Edition of the American Joint Committee on Cancer (AJCC) staging standards. The overall survival (OS) was defined as the time from the date of surgery to the date of death.

### Mismatch repair (MMR) status

Samples were stratified according to DNA MMR status, as described previously^28^. Briefly, paraffin sections were baked overnight, deparaffinized in xylene, rehydrated through graded ethanol, quenched for endogenous peroxidase activity in hydrogen peroxide and incubated with primary antibodies. Then, the sections were incubated with the primary antibody overnight and stained with diaminobenzidine. MMR-proficient tumors were defined as those simultaneously expressing MutL homolog 1 (MLH1), MutS homolog 2 (MSH2), PMS1 Homolog 2 (PMS2) and MutS homolog 6 (MSH6), whereas MMR-deficient tumors were defined as those lacking the expression of at least one of these markers.

### Immunohistochemistry

We constructed a tissue microarray (TMA) from colon cancer blocks and performed an immunohistochemistry (IHC) analysis. IHC staining was performed according to the standard protocols. Siglec15 rabbit antibody (1:500; Novus; NBP2-41162), PD-L1 rabbit antibody (1:50; Cell Signaling Technology; CST #13684), CD8 rabbit antibody (1:50; Cell Signaling Technology; CST#85336) and CD4 (ZSGB-BIO; ZM-0418) were used for immunostaining. To score tumor cells as positive, both nuclear and cytoplasmic staining were counted. For quantitative analysis, the histochemistry score (H-score) was calculated based on the staining intensity and percentage of stained cells using the HALO image analysis platform. The intensity score was defined as follows: 0, no appreciable staining in cells; 1, weak staining in cells comparable to that in stromal cells; 2, intermediate staining; and 3, strong staining. The fraction of positive cells was scored as 0%-100%. The H-score was calculated by multiplying the intensity and fraction scores using the following formula:

[1× (% cells 1+) + 2× (% cells 2+) + 3× (% cells 3+)]

with a total range of 0-300. Tissue sections were examined and scored separately by two independent investigators who were blind to the clinicopathological data.

The Siglec15 and PD-L1 expression levels on TA and SA were evaluated by H-score. Siglec15 expression was evaluated based on immunostaining in the cytoplasm and membrane of cells, and the expression level (intensity) was scored as 0 (absent), 1 (weak), 2 (intermediate) or 3 (strong). The expression of Siglec15 was evaluated in the whole tumor tissue (whole), TA, SA cells on stained sections (**Figure S1**), and the median H-score was chosen as the cut-off value to define as high and low (<70, Siglec15(whole)^Low^ and ≥70, Siglec15(whole)^High^; <120, Siglec15(TA)^Low^ and ≥120, Siglec15(TA)^High^; <35, Siglec15(SA)^Low^ and ≥35, Siglec15(SA)^High^; respectively). When detecting Siglec15 expression in SA, we analyzed all kinds of cells in stromal without selection (the green region). To some extent, we explored Siglec15 expression in TA and tumor infiltrating myeloid cells, respectively. Here, we are unable to distinguish the type of cells in stromal region without staining specified markers. The expression of PD-L1 was also evaluated in the whole tumor tissue (whole), TA, SA cells, and was defined as positive when PD-L1 staining was present on ≥1% of cells 26, 29. For CD8 and CD4 evaluation, the number of CD8^+^ or CD4^+^ T cell in SA was counted, and the density (cells/mm^2^) of each T-cell population in the SA was determined.

### Statistical analysis

Correlation analyses were performed using the Student's *t*-test and χ2 test, whereas survival analysis was performed using the Kaplan-Meier method to depict the survival curves of OS. A log-rank test was performed to examine intergroup differences. Univariate and multivariate analyses were performed using the Cox proportional hazard model. All statistical analyses were accomplished by SPSS software (version 26.0) and GraphPad Prism 8 software (La Jolla, California, USA). Statistical significance was set at *p* < 0.05. * *p* < 0.05, ** *p* < 0.01, and *** *p* < 0.001.

## Results

### Association of Siglec15 expression with the clinicopathological characteristics

The clinicopathological characteristics of the COAD cohort were illustrated (**Table S1**). In this study, the median age of all COAD patients was 68 years (range from 24-90 years) and 58 (56.9 %) and 44 (43.1 %) were male and female, respectively. The median overall survival (OS) time was 51 months (range from 1-108 months) and 61 patients died during the follow-up.

**Figure 1:**
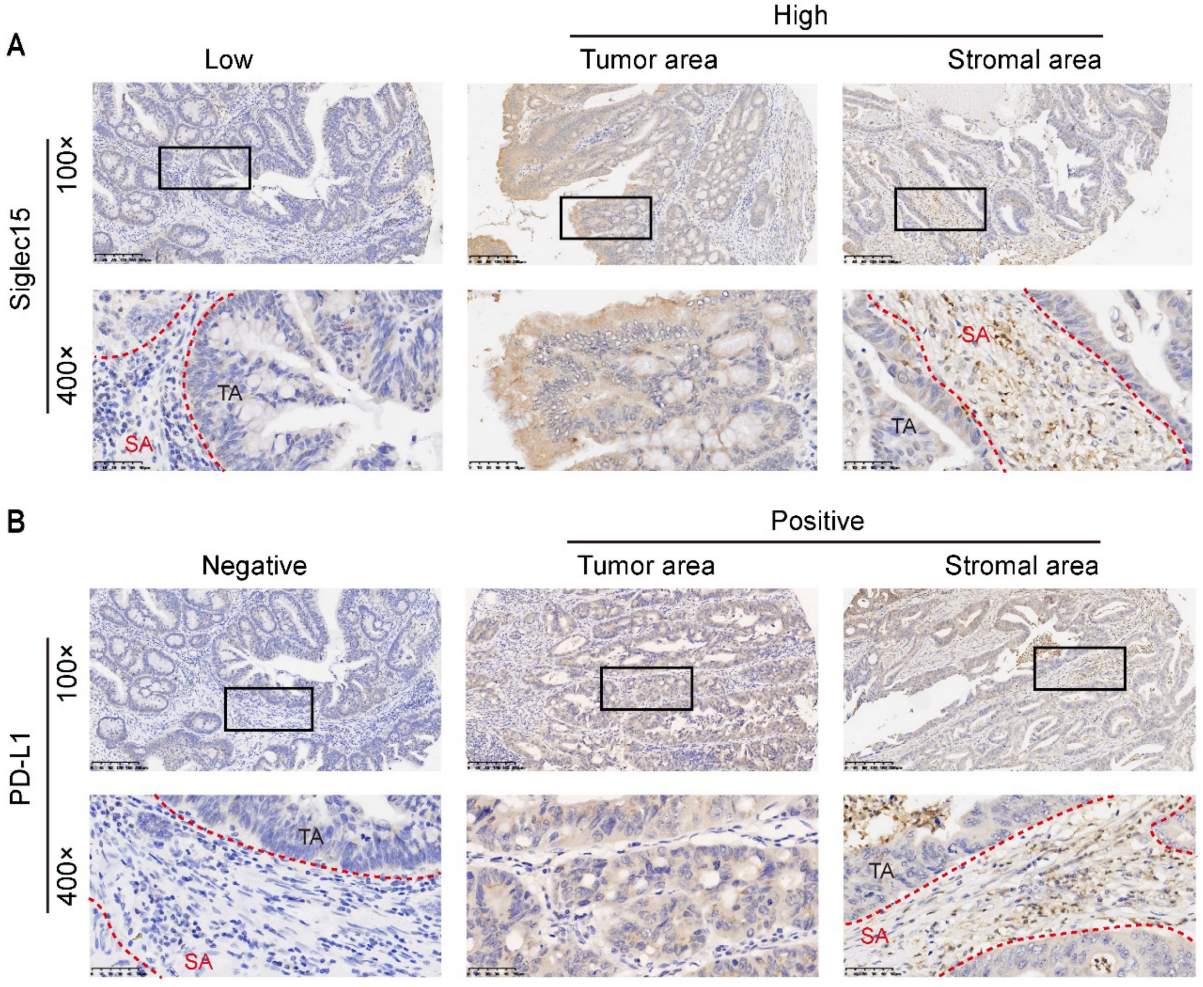
Siglec15 and PD-L1 expression in COAD cancer samples. Representative micrographs of Siglec15 (A) and PD-L1 (B) expression within the stromal area (SA) and tumor area (TA). Representative images are presented in 100× (upper) and 400× (below).

As Siglec15 is broadly upregulated in human cancer cells and tumor infiltrating myeloid cells, we detected the Siglec15 expression in TA, SA and whole cells. The same method was used to detect the PD-L1 expression in colon cancer (**Figure 1**). The positive rates of Siglec15 expression were 55.9% (57/102 in SA), 65.7% (67/102 in TA) and 58.8% (60/102 in whole), respectively (**Table 1 and Supplementary Table S1**).

Next, we explored the association between Siglec15 expression and clinicopathological characteristics. No significant associations were found between Siglec15 expression and clinicopathological characteristics, such as age, gender, tumor location, TNM stage, MMR status, and tumor growth pattern, excluding that Siglec15(SA) was correlated with age (p<0.05) (**Table 1**).

### Prognostic significance of Siglec15 and PD-L1 on the overall survival

The univariate Cox regression analyses of patients revealed that age (HR=1.947, 95% CI: 1.152 to 3.291, p<0.01), TNM stage (HR=2.910, 95% CI: 1.746 to 4.850, p<0.001), PD-L1(SA) (HR=0.493, 95% CI: 0.278 to 0.874, p=0.015) and Siglec15(SA) (HR=2.481, 95% CI: 1.450 to 4.245, p<0.01) expression were associated with prognosis of COAD patients in terms of OS (**Table 2**). Multivariate Cox regression analysis indicated that only TNM stage (HR=3.059, 95% CI: 1.799 to 5.200, p<0.001), PD-L1(SA) (HR=0.352, 95% CI: 0.193 to 0.642, p=0.001) and Siglec15(SA) (HR=3.264, 95% CI: 1.808 to 5.890, p<0.001) expression were independent prognostic factors for OS in COAD patients, but not Siglec15(whole) and Siglec15(TA) (**Table 2**).

**Figure 2:**
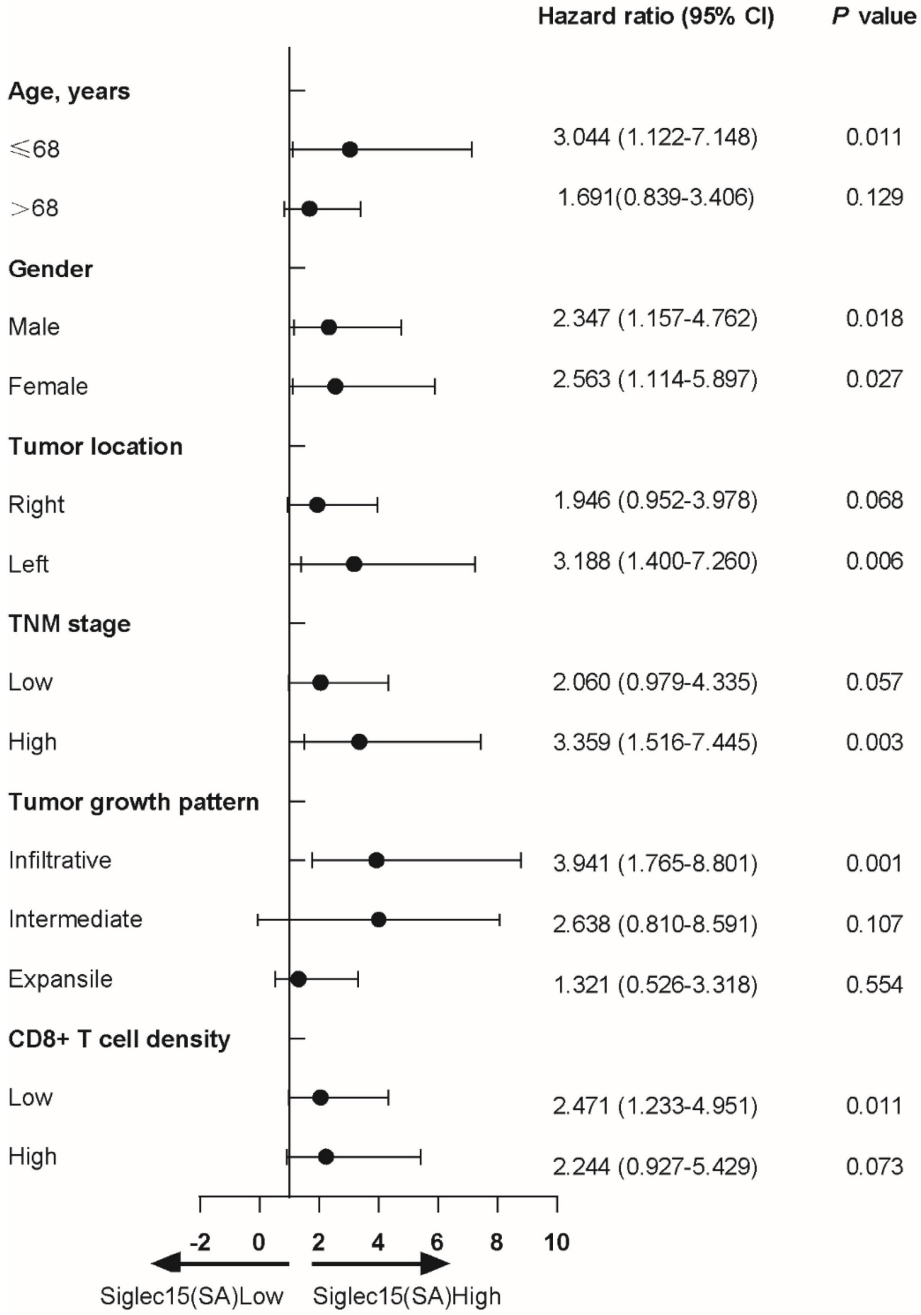

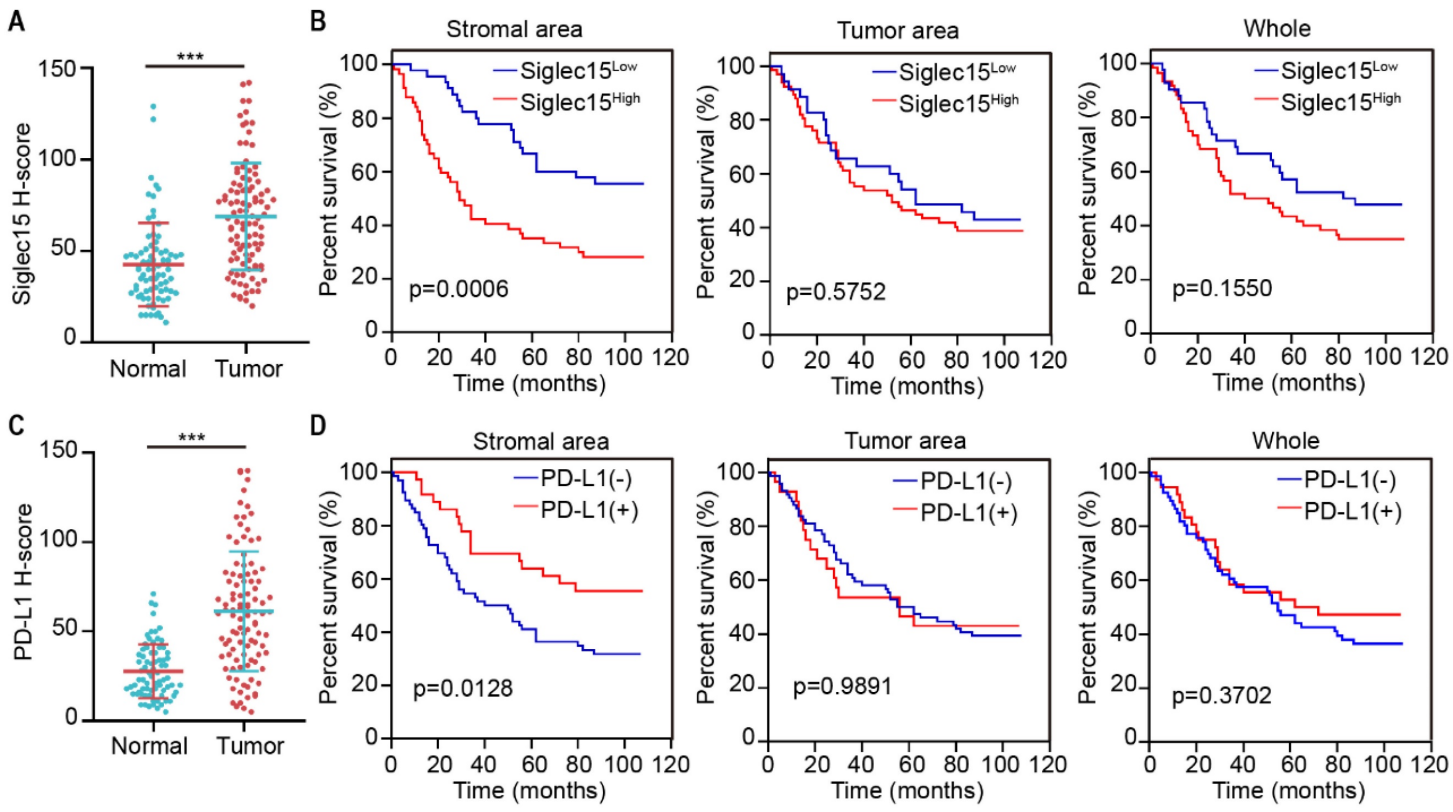
Prognostic significance of Siglec15 and PD-L1 expression on prognosis in COAD. (A) H-score of Siglec15 in the primary human COAD tissue microarray and adjacent normal tissues. Data are means ± SEM. p-values are according to Student's *t*-test. (B) Overall survival curves were generated based on the protein levels of Siglec15 in stromal area, tumor area and whole cells of COAD. (C) H-score of PD-L1 in the primary human COAD tissue microarray and adjacent normal tissues. Data are means ± SEM. p-values are according to Student's *t*-test. (D) Overall survival curves were generated based on the protein levels of PD-L1 in stromal area, tumor area and whole cells of COAD. p-values are according to Kaplan-Meier plots and are compared to the log-rank test.

Next, we analyzed the protein expression of Siglec15 in patients with COAD. Compared to adjacent normal tissue, Siglec15 expression was significantly higher in tumors (**Figure 2A**). Kaplan-Meier analysis also suggested that Siglec15(SA)^High^ was significantly associated with poor OS (p<0.001), but not Siglec15(whole)^High^ and Siglec15(TA)^High^ (**Figure 2B**). We also examined the relationship of Siglec15(SA) expression levels with clinicopathological characteristics. Siglec15(SA) expression were poor prognosis for patients—under 68 years old, male, left side tumor, high stage, infiltrative tumor, and low CD8^+^ T cell density (**Table 3**).

PD-L1 expression was significantly higher in the tumor tissues than in the normal tissues (**Figure 2C**). Kaplan-Meier analysis suggested that patients with PD-L1(SA)^+^ had longer OS than those with PD-L1(SA)^-^; however, there were no significant differences in survival between patients with PD-L1(whole)^+^ and those with PD-L1(whole)^-^, or between patients with PD-L1(TA)^+^ and those with PD-L1(TA)^-^ (**Figure 2D**).

### Relationship between Siglec15 or PD-L1 and infiltration of T cells in COAD

**Figure 3:**
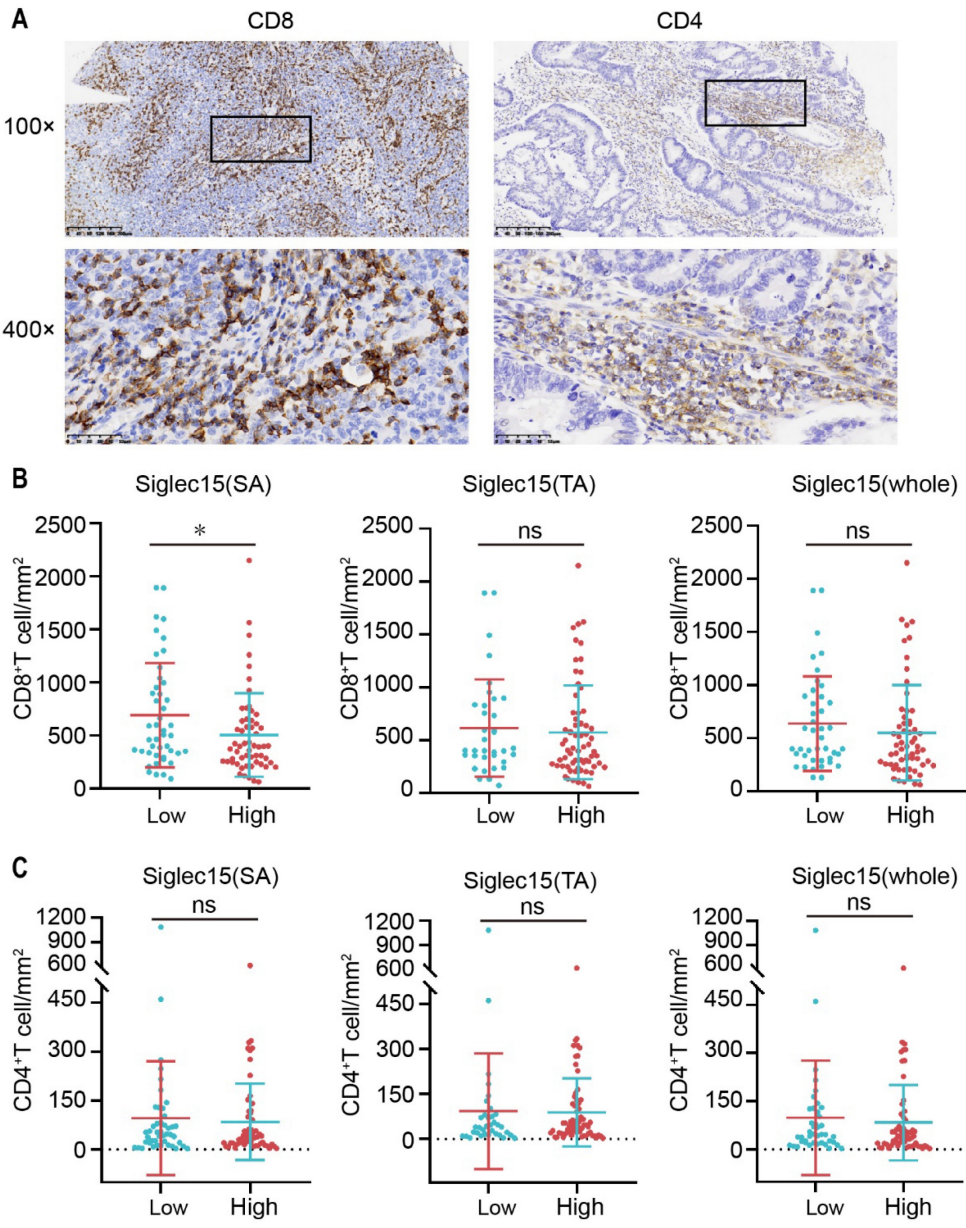
Relationship between Siglec15 expression and the infiltration of CD4+/CD8+ TILs. (A) Representative micrographs of CD8 (left) and CD4 (right) expression in the COAD. Representative images are presented in 100× (upper) and 400× (below). (B and C) The correlation of Siglec15 expression and CD8^+^ T cells (B) and CD4^+^ T cells (C) in the COAD. Data are means ± SEM. p-values are according to Student's *t*-test.

As the Siglec15 and PD-L1 expression levels influence the tumor microenvironment 15, 30, we were curious regarding CD4^+^ and CD8^+^ tumor infiltrating lymphocytes (TILs) in COAD (**Figure 3A**). To assess the number of CD4^+^ and CD8^+^ lymphocytes, we classified tissues into TA and SA and counted the number of CD4^+^ and CD8^+^ TILs in SA, which were classified to be positive or negative (**Figure S1B**). As shown, the density of CD8^+^ TILs was inversely associated with the expression level of Siglec15(SA), but was not significantly associated with the expression levels of Siglec15(TA) and Siglec15(whole) in COAD (**Figure 3B**). Regarding CD4^+^ TILs, there was no significant association between them and the Siglec15 expression level (**Figure 3C**). In addition, the densities of CD4^+^ and CD8^+^ TILs were not associated with PD-L1 expression (**Figure S2, A and B**).

### Significance of Siglec15 and PD-L1 in the MMR-proficient COAD

**Figure 4:**
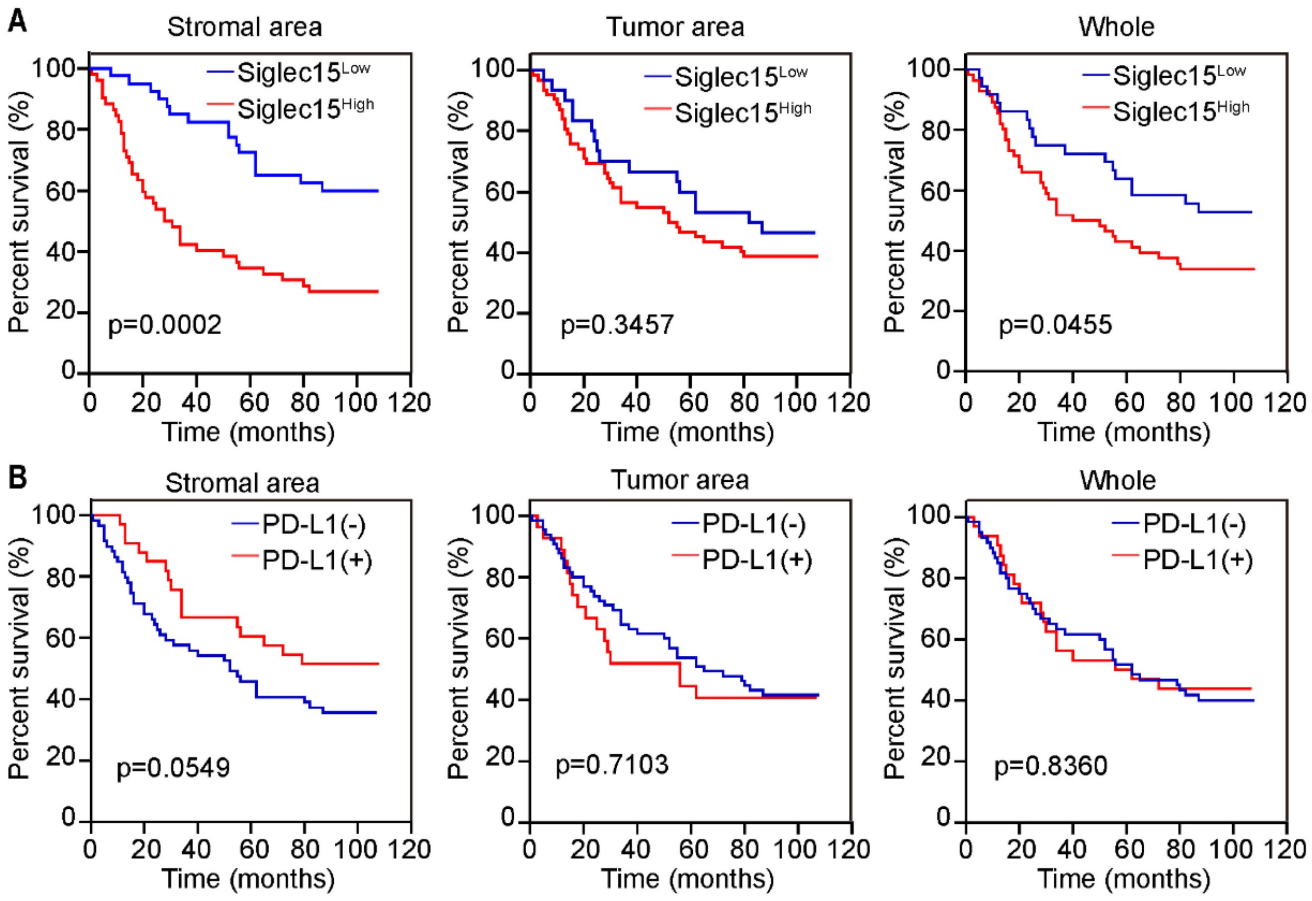
Prognostic significance of Siglec15 and PD-L1 in MMR-p COAD. (A and B) Prognostic significance of Siglec15 (A) and PD-L1 (B) in stromal area, tumor area and whole cells of MMR-p COAD, respectively. p-values are according to Kaplan-Meier plots and compared to the log-rank test.

Previous studies have indicated that patients with MMR-p COAD could not benefit from immunotherapy; therefore, we were curious regarding the role of Siglec15 and PD-L1 in MMR-p colon cancer. Kaplan-Meier analyses revealed that patients with high Siglec15(SA) levels had a significantly shorter OS (p<0.05) than those with low Siglec15(SA) levels in MMR-p COAD. Notably, Siglec15(whole), a factor without a significant prognostic value in all patients with COAD, showed a significant predictive value for the survival of patients with MMR-p COAD (**Figure 4A**). All types of PD-L1 expression status were not correlated with the survival of patients with MMR-p COAD (**Figure 4B**).

### Association of Siglec15 and PD-L1 in COAD

**Figure 5:**
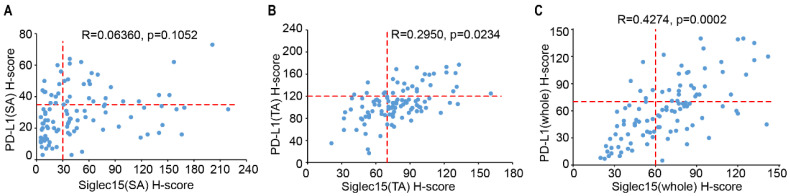
The association of Siglec15 and PD-L1 in COAD. (A to C) The correlation between Siglec15 and PD-L1 expression was assessed in stromal area (A), tumor area (B) and whole cells (C) on COAD tissue microarray. The Pearson *R* scores and p-values are shown.

As Siglec15 is mutually exclusive to PD-L1, we analyzed the association between Siglec15 and PD-L1 expression in TA, SA and whole cells. In 102 samples, there was no correlation between Siglec15(SA) and PD-L1(SA) expression (R=0.06360, p=0.1052) (**Figure 5A**), with 12 of the Siglec15(SA)^Low^ patients (33.3%) being PD-L1(SA)^+^, and 33 of the PD-L1(SA)^-^ patients (50.0%) being Siglec15(SA)^High^. Similarly, there was no correlation between Siglec15(TA) and PD-L1(TA) expression (R=0.2950, p=0.0234) (**Figure 5B**). However, there was positive correlations between Siglec15(whole) and PD-L1(whole) expression (R=0.4274, p=0.0002) (**Figure 5C**). These data suggested that there were no correlations between Siglec15(SA) and PD-L1(SA), and between Siglec15(TA) and PD-L1(TA) in COAD, and there was positive correlation between Siglec15(whole) and PD-L1(whole).

### Immune classification of Siglec15 and PD-L1 in COAD

**Figure 6:**
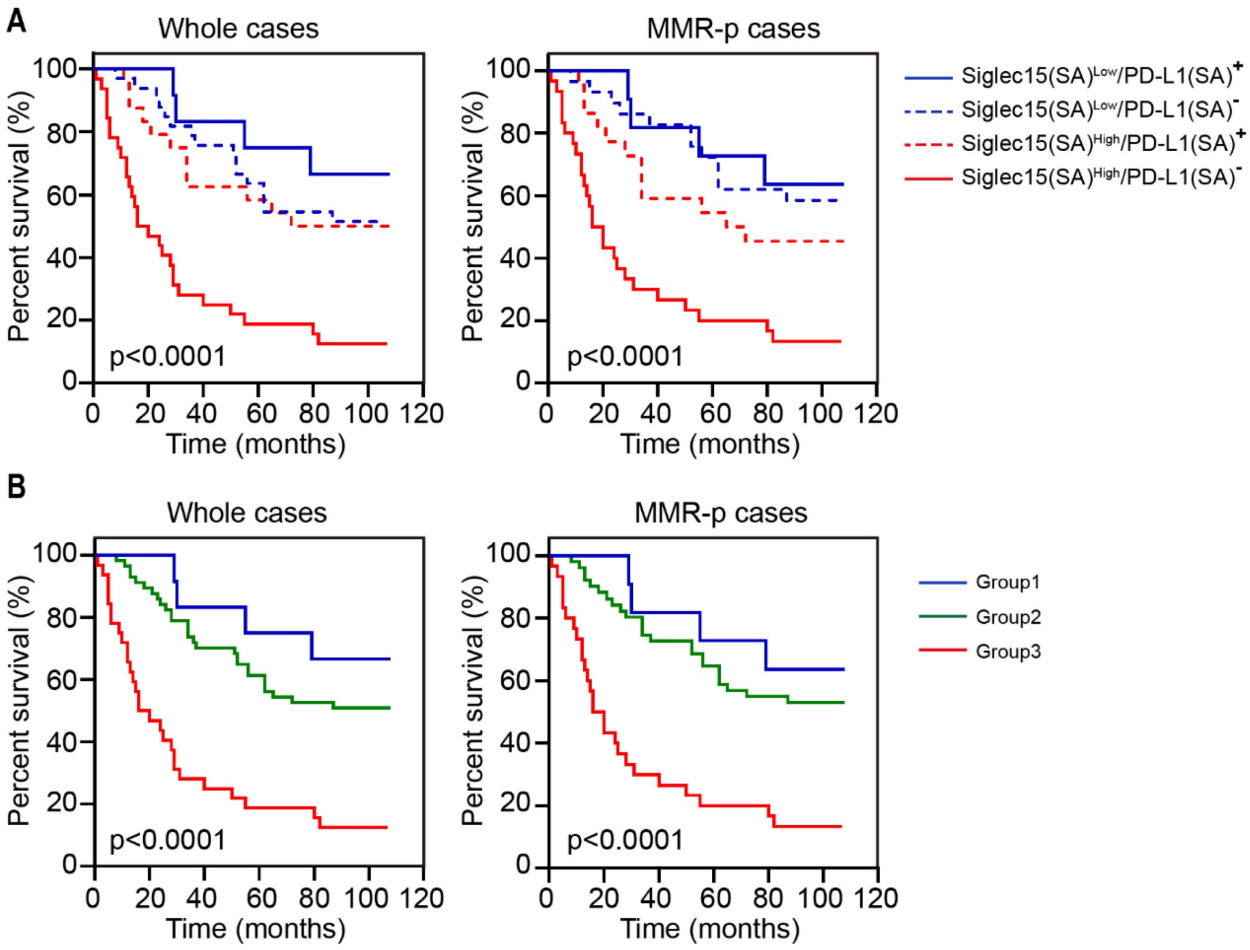
Survival analysis for patients with COAD based on Siglec15 and PD-L1 expression in stromal area. (A) Kaplan-Meier survival curves for overall survival based on the expression status of Siglec15(SA)/PD-L1(SA) in COAD and MMR-p COAD. (B) Kaplan-Meier survival curves for OS in three groups: group 1, Siglec15(SA)^Low^ /PD-L1(SA)^+^; group 2, Siglec15(SA)^Low^/PD-L1(SA)^-^ or Siglec15(SA)^High^/PD-L1(SA)^+^ and group 3, Siglec15(SA)^High^/PD-L1(SA)^-^. *p*-values using Kaplan-Meier plots and compared with the log-rank test.

Given that Siglec15(SA) and PD-L1(SA) were significantly associated with OS, we explored the expression of Siglec15(SA) and PD-L1(SA) and analyzed their association with the survival of patients with COAD. Kaplan-Meier analysis demonstrated that patients with Siglec15(SA)^Low^/PD-L1(SA)^+^ had the best prognosis and those with Siglec15(SA)^High^/PD-L1(SA)^-^ had the worst prognosis, both in MMR-p COAD and the whole COAD tissue (**Figure 6A**). Next, we classified the patients into three groups: group 1 (Siglec15(SA)^Low^/PD-L1(SA)^+^), group 2 (Siglec15(SA)^Low^/PD-L1(SA)^-^ or Siglec15(SA)^High^/PD-L1(SA)^+^), group 3 (Siglec15(SA)^High^/PD-L1(SA)^-^), and group 1 was with low risk, group 2 with moderate risk and group 3 with high risk, both in MMR-p COAD and the whole COAD (**Figure 6B**).

## Discussion

Siglec15, a novel immune checkpoint molecule, is an emerging target for next-generation cancer immunotherapy. In this study, we first found that Siglec15 protein levels were higher in tumor tissues than in normal tissues, and elevated Siglec15(SA)— but not Siglec15(TA) and Siglec15(whole)—correlated with poor prognosis in COAD and MMR-p COAD and served as an immune suppressor by suppressing CD8^+^ T cell, but not CD4^+^ T cell. In addition, there were no correlations between Siglec15 and PD-L1 in SA and TA, whereas Siglec15 was correlated with PD-L1 in whole COAD tissues.

Based on pan-cancer analysis from The Cancer Genome Atlas database, Siglec15 was upregulated across most cancer types, including COAD^23^, which was consistent with our results by detecting protein expression. Considering Siglec15 is expressed in tumor cells and tumor infiltrating myeloid cells, we detected Siglec15 in SA, TA and whole cells. Our results showed that Siglec15(SA)^High^, rather than Siglec15(TA)^High^ and Siglec15(whole)^High^, were correlated with poor prognosis.

Furthermore, the protein level of Siglec15 was positively correlated with that of PD-L1 in TA and whole cells. Siglec15 is broadly expression on human cancer cells and tumor infiltrating myeloid cells, and it has been reported that SIGLEC15^+^ tumor-associated macrophage, rather than SIGLEC15^+^ PDAC cells, correlated with poor prognosis in PDAC^31^. Different cells expressing Siglec15 may execute different functions, and it's important to classify Siglec15 expression. Tumor Siglec15 can be downregulated by IFN-γ, which is the dominant cytokine required for PD-L1 induction^15^. Moreover, it was reported that higher Siglec15 expression in EGFR-mutant lung cancers and EGFR-mutant lung cancer cells induced the expression of PD-L1^32^, ^33^. These contradictory results in different types of cancer reveal that the complexity and heterogeneity in tumors and that Siglec15 and PD-L1 may be simultaneously regulated by other molecules in COAD; this line of research needs to be further investigated in future studies.

In addition, the expression of PD-L1 in TA or whole cells was not correlated with COAD patients' prognosis, which is consistent with previous large sample study^26^. In accordance with a previous study^34^, patients with PD-L1(SA)^+^ had longer survival in COAD in our study. However, PD-L1(SA) expression was not correlated with MMR-p COAD patient prognosis, whereas Siglec15(SA)^High^ was correlated with poor prognosis in MMR-p COAD. These data indicated that Siglec15(SA) may be an immune biomarker of MMR-p COAD. MMR-P COAD is considered as immune desert tumor, and several clinical trials have revealed that no responses were observed among the patients with MMR-p tumors treated with PD-L1 inhibitors. Similarly, Siglec15 was also overexpression in immune desert tumor—sarcoma, and was involved in immune-related pathways and predicted poor prognosis^23^. Our results indicated that Siglec15 may be an immune predicative biomarker in MMR-p COAD and combining Siglec15 antibody with PD-1/PD-L1 antibodies may benefit MMR-p COAD patients, which require further investigation and clinical trials to confirm.

Currently, the relationship between Siglec15 expression and immune cell infiltration in different types of cancers remains uncertain. Chen *et al.* demonstrated that Siglec15 on macrophages/myeloid cells suppresses CD8^+^ T cell responses^15^. Yang *et al.* reported that Siglec15 is significantly associated with Treg infiltration in cancers and positively associated with Foxp3 in LUAD^23^. Chen *et al.* found that Siglec15 expression in tumor cells was negatively correlated with the density of CD45RO T cells and Tregs and had no effect on the density of macrophage in PDCA^22^. Zu *et al.* reported that Siglec15 was negatively correlated with the infiltration levels of CD8^+^ T cells, natural killer cells, macrophages, type 1 T helper cells, and dendritic cells in bladder cancer^21^. In CRC sentinel lymph nodes, Siglec15 expression suppressed T-cell responses^35^. In this study, we found that Siglec15(SA)^High^ was significantly associated with a low density of CD8^+^ TILs, but not with the infiltration of CD4^+^ TILs in COAD. Our data suggested that Siglec15(SA) expression suppressed immune by reducing CD8^+^ TILs in COAD. Similar phenomena were observed in MMR-p COAD. Interestingly, we observed a higher density of CD4^+^ and CD8^+^ T cells in normal tissues than in tumors (Figure S4). Siglec15(SA) may play a role in immunosuppression in MMR-p COAD by inhibiting T-cell proliferation. A possible mechanism is that Siglec15(SA)^High^ cells are continuous secreting some inhibitory cytokines inhibiting T-cell proliferation, which needs further study. If the treatment with Siglec15 antibody could recruit more CD8^+^ T cells to the tumor, it may convert the MMR-p COAD from a “cold” to a “hot” tumor.

However, there are also some limitations in our study. First, it was a retrospective study in a medium sample size and a single central cohort; thus, selection bias was inevitable, which may limit the generalizability of results. Second, we evaluated the Siglec15 and PD-L1 expression and T-cell markers such as CD4 and CD8 using TMA IHC and focused on information within SA, which may have ignored some other information regarding the tumor. Therefore, clinical data from multicenter and prospective studies are required for further research.

In conclusion, this study showed that Siglec15 expression in COAD tissues was higher than in normal tissues, and high Siglec15 expression in SA, rather than in TA and whole cells, indicated poor prognosis by suppressing immune responses by reducing the density of CD8^+^ TILs in COAD and MMR-p COAD. These results may provide new insights into patients with COAD or MMR-p COAD when choosing appropriate immunotherapy.

## Supplementary Material

Supplementary figures and tables.

## Figures and Tables

**Table 1:** The association of Siglec15 expression with clinicopathological characteristics

		Siglec15(whole)			Siglec15(TA)			Siglec15(SA)		
	Patients	Low	High	p value	Low	High	p value	Low	High	p value
Age (median 68)				0.6185			0.6739			0.0064
≤68	48	21	27		16	32		28	20	
>68	54	21	33		19	35		17	37	
Gender				0.1148			0.5718			0.5225
Male	58	20	38		19	39		24	34	
Female	44	22	22		16	28		21	23	
Tumor location				0.9433			0.8886			0.5185
Right	49	20	29		18	31		20	29	
Left	53	22	31		17	36		25	28	
Tumor growth pattern				0.5059			0.442			0.5355
Expansile	33	16	17		12	21		12	21	
Intermediate	24	8	16		7	17		11	13	
Infiltrative	45	18	27		16	29		22	23	
TNM stage				0.4216			0.6656			0.3654
Low	63	24	39		22	41		30	33	
High	39	18	21		13	26		15	24	

**Table 2:** Univariate and multivariate Cox proportional hazards analysis of OS in COAD patients

Variables	Univariable OR (95% CI)	p Value	Multivariable OR (95% CI)	p Value
Age (>68/≤68)	1.947 (1.152 to 3.291)	0.013	1.502 (0.869 to 2.595)	0.145
Gender (Male/Female)	1.182 (0.707 to 1.977)	0.524		
Tumor location (Right/Left)	1.560 (0.942 to 2.584)	0.084		
Tumor growth pattern				
Infiltrative/Expansile	1.335 (0.664 to 2.685)	0.418		
Intermediate/Expansile	1.448 (0.750 to 2.797)	0.270		
TNM stage (High/Low)	2.910 (1.746 to 4.850)	**<0.0001**	3.059 (1.799 to 5.200)	**<0.0001**
CD8^+^ T cell density (High/Low)	0.736 (0.431 to 1.256)	0.261		
PD-L1 (SA) (Positive/Negative)	0.493 (0.278 to 0.874)	**0.015**	0.352 (0.193 to 0.642)	**0.001**
PD-L1 (TA) (Positive/Negative)	0.996 (0.563 to 1.763)	0.989		
PD-L1 (whole) (Positive/Negative)	0.782 (0.455 to 1.345)	0.374		
Siglec15 (SA) (High/Low)	2.481 (1.450 to 4.245)	**0.001**	3.264(1.808 to 5.890)	**<0.0001**
Siglec15 (TA) (High/Low)	1.164 (0.682 to 1.987)	0.578		
Siglec15 (whole) (High/Low)	1.455 (0.862 to 2.456)	0.160		

**Table 3:** Cox regression analysis to assess the relationship of Siglec15(SA) expression levels with clinicopathological characteristics
