# Supplementary material for: Tumor stroma Siglec15 expression is a poor prognosis predictor in colon adenocarcinoma
Source: J Cancer. 2023 Sep 18;14(16):2998–3008. doi: 10.7150/jca.87618 (PMC10583595; doi:10.7150/jca.87618)
Supplement: Supplementary file 1 — Supplementary figures and tables. [file jcav14p2998s1.pdf]

Supplementary Materials for

Tumor stroma Siglec15 expression is a poor prognosis predictor in colon adenocarcinoma

Authors:

Weixiang Zhan[1,2,3†], Fan Bai[1,2,3†], Yue Cai[1,2,3†], Jianwei Zhang[1,2,3], Ge Qin[1,2,3], Yuqian Xie[1,2,3], and Yanhong Deng[1,2,3*]

† **These authors have contributed equally to this work**

* **Correspondence to:**

Yanhong Deng (Email: dengyanh@mail.sysu.edu.cn; Phone: +86(0)20-38254084)

This PDF file includes:

Figures S1 to S4
Tables S1 to S2

Table S1. Basic characteristics of patients in colon adenocarcinoma

Characteristic	Patients
Age (Median 68)	
Mean (Range)	68 (24-90)
≤68	54 (52.9%)
> 68	48 (47.1%)
Gender	
Male	58 (56.9%)
Female	44 (43.1%)
Tumor location	
Right	49 (48.0%)
Left	53 (52.0%)
Tumor growth pattern	
Expansile	33 (32.4%)
Intermediate	24 (23.5%)
Infiltrative	45 (44.1%)
T classification (TNM)	
T1~2	5 (4.9%)
T3	84 (82.4%)
T4	13 (12.7%)
N classification (TNM)	
N0	63 (61.8%)
N+	39 (38.2%)
M classification (TNM)	
M0	99 (97.1%)
M1	3 (2.9%)
MMR status	
MMR-proficient	92 (90.2%)
MMR-deficient	10 (9.8%)
Siglec15 (SA)	
Low	45 (44.1%)
High	57 (55.9%)
Siglec15 (TA)	
Low	35 (34.3%)
High	67 (65.7%)
Siglec15 (whole)	
Low	42 (41.2%)
High	60 (58.8%)

Table S2. The association of Siglec15 expression with clinicopathological characteristics in MMR-p COAD

	Patients	Siglec15(whole)		p value	Siglec15(TA)		p value	Siglec15(SA)		p value
		Low	High		Low	High		Low	High	
Age (median 68)				0.6152			0.6488			0.0079
≤68	43	18	25		13	30		25	18	
> 68	49	18	31		17	32		15	34	
Gender				0.106			0.8988			0.657
Male	53	17	36		17	36		22	31	
Female	39	19	20		13	26		18	21	
Tumor location				0.9851			0.4657			0.4397
Right	41	16	25		15	26		16	25	
Left	51	20	31		15	36		24	27	
Tumor growth pattern				0.7193			0.914			0.4374
Expansile	27	12	15		9	18		9	18	
Intermediate	24	8	16		7	17		11	13	
Infiltrative	41	16	25		14	27		20	21	
TNM stage				0.2329			0.8347			0.4373
Low	58	20	38		20	38		27	31	
High	34	16	18		10	24		13	21	

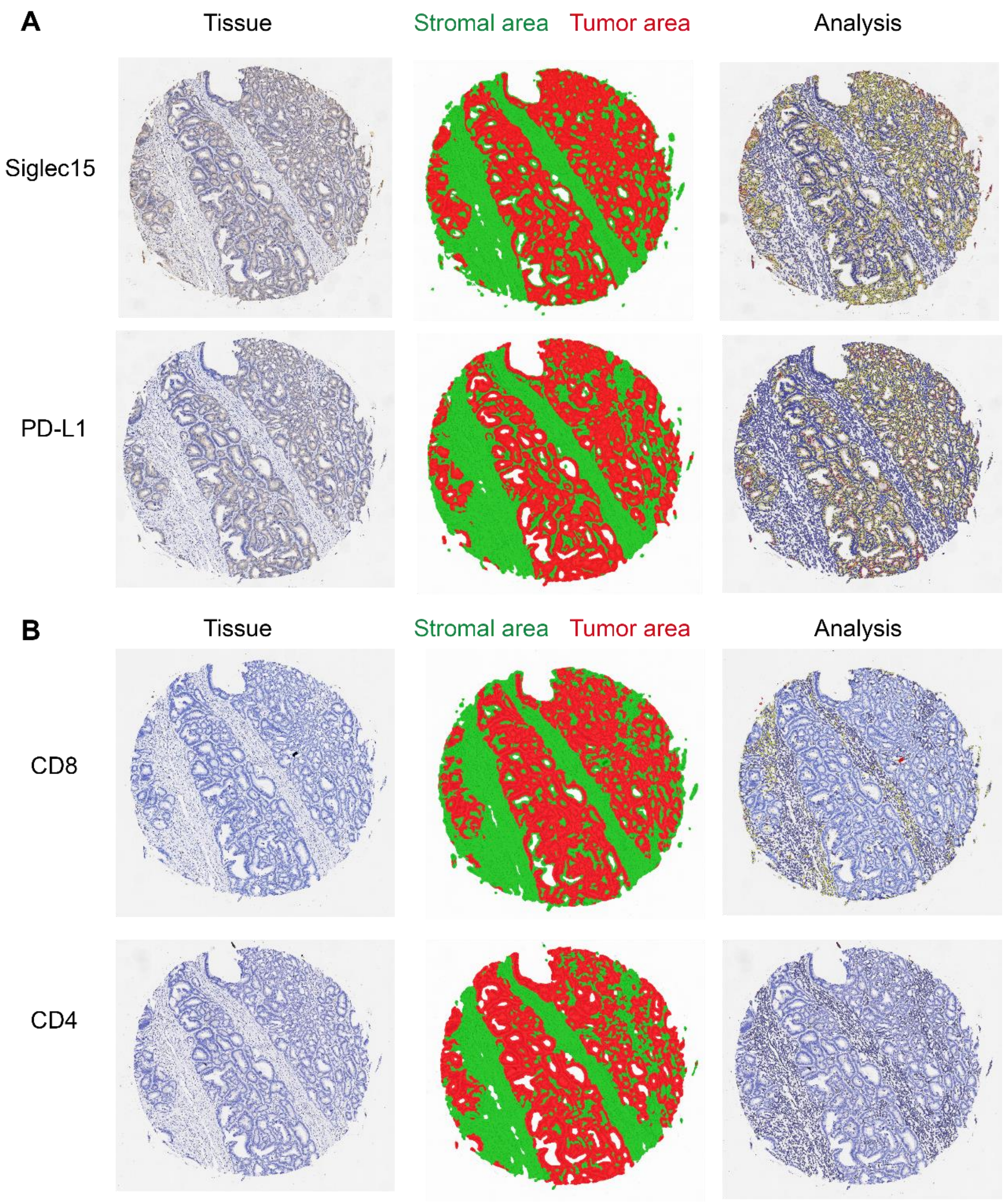

Figure S1 The classification of tissue into stromal area and tumor area. (A) Micrographs of Siglec15 and PD-L1 expression within tissue (left), and the classified stromal area and tumor area (middle), and the analysis of tumor or stromal area (right). (B) Micrographs of CD4 and CD8 expression within tissue(left), and the classified stromal area and tumor area (middle), and the analysis of stromal area(right).

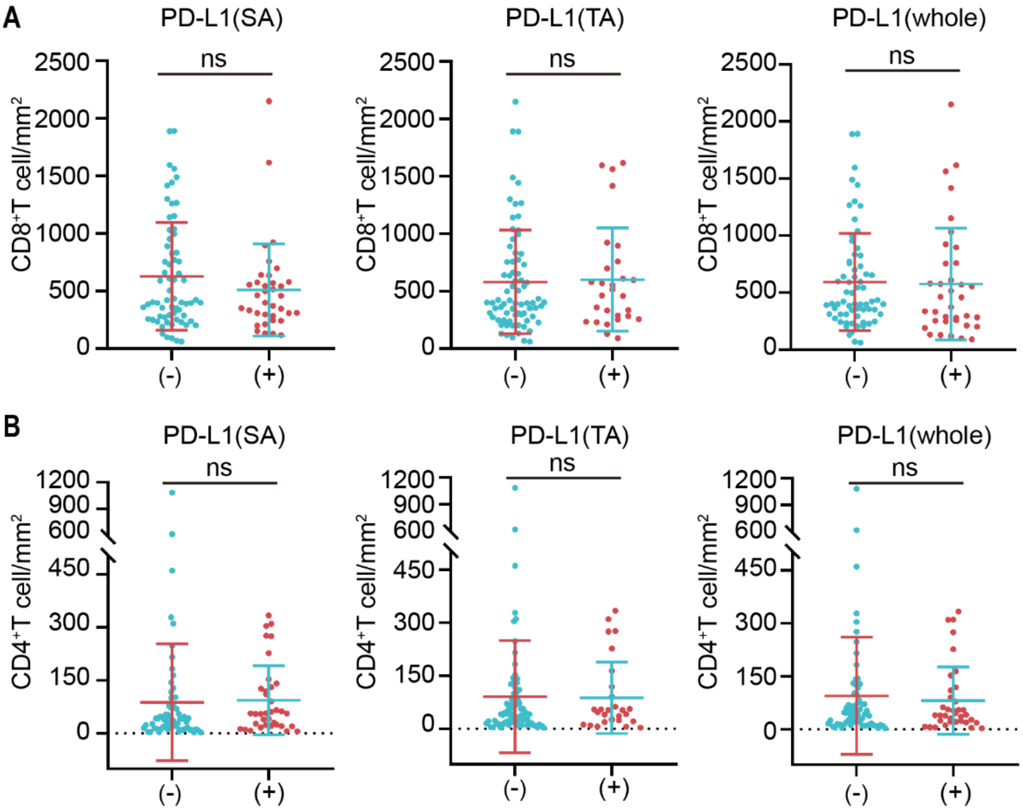

Figure S2 Relationship between PD-L1 expression and the infiltration of CD4+/CD8+ TILs. (A and B) The correlation of PD-L1 expression and CD8+ T cells (A) and CD4+ T cells (B) in the COAD. Data are means ± s.e.m. p values by student's *t*-test.

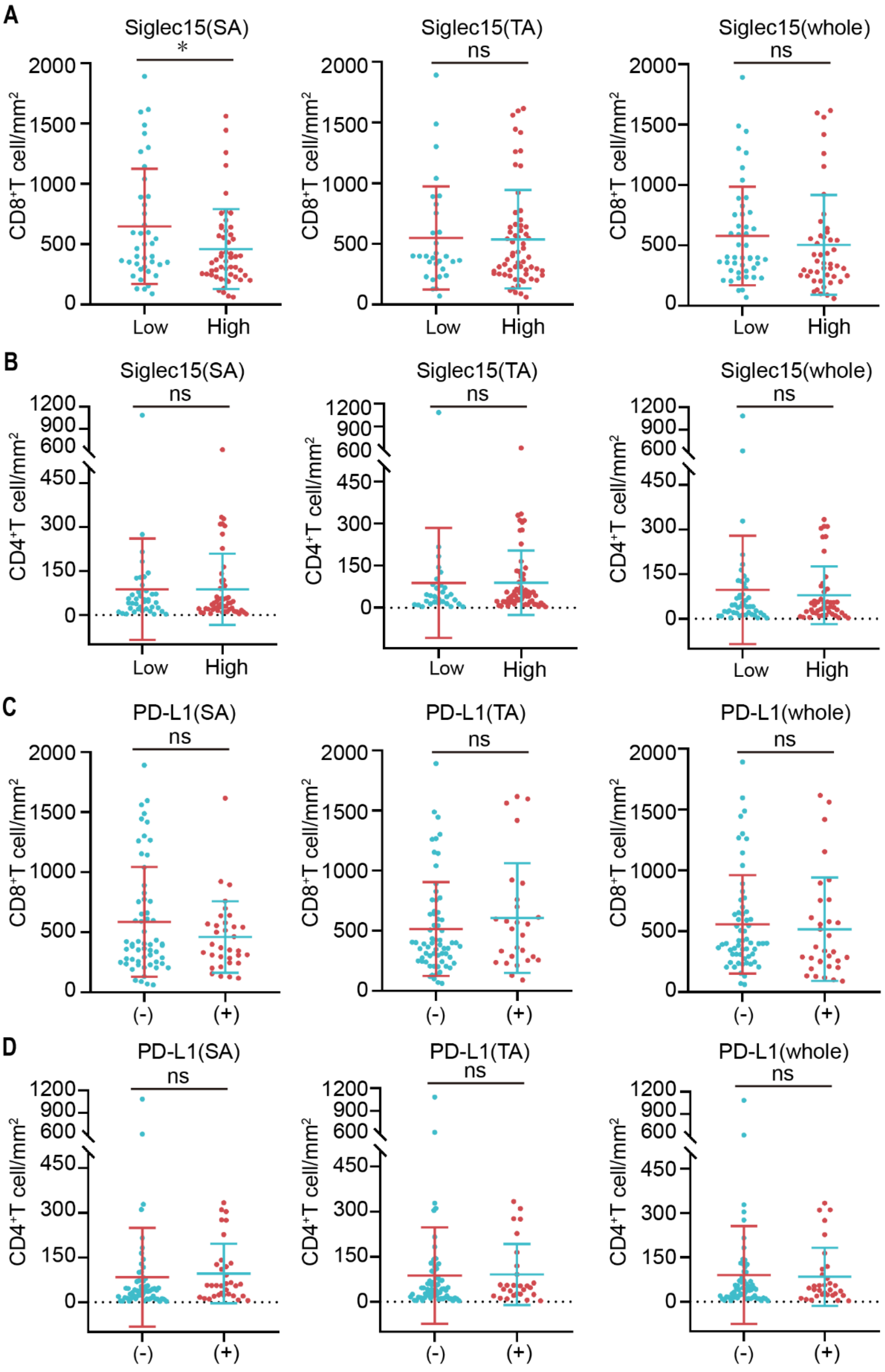

Figure S3 The correlation of Siglec15 and PD-L1 expression and $CD8^+$ T cells and $CD4^+$ T cells in the MMR-p COAD. (A and B) The correlation of Siglec15 expression and $CD8^+$ T cells (A) and $CD4^+$ T cells (B) in the MMR-p COAD. (C and D) The correlation of PD-L1 expression and $CD8^+$ T cells (C) and $CD4^+$ T cells (D) in the MMR-p COAD. Data are means ± s.e.m. p values by student's *t*-test.

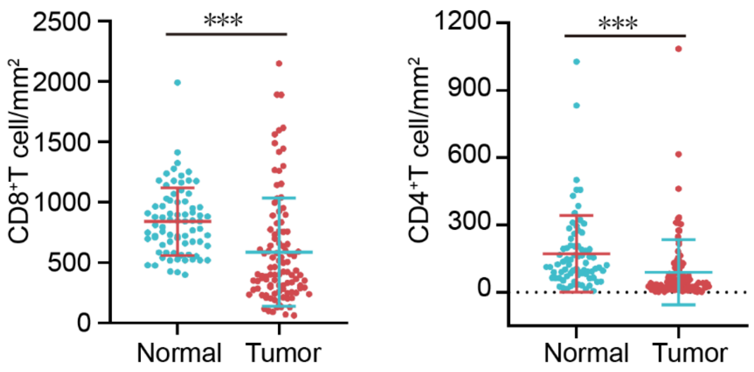

Figure S4 The comparation of CD8^{+} T cells and CD4^{+} T cells between COAD and adjacent normal tissues. Data are means ± s.e.m. p values by student's *t*-test.
